# miR-21 promotes non-small cell lung cancer cells growth by regulating fatty acid metabolism

**DOI:** 10.1186/s12935-019-0941-8

**Published:** 2019-08-23

**Authors:** Kewei Ni, Dimin Wang, Heyun Xu, Fuyang Mei, Changhao Wu, Zhifang Liu, Bing Zhou

**Affiliations:** 1 Department of Cardiothoracic Surgery, Zhejiang Provincial People’s Hospital, People’s Hospital of Hangzhou Medical College, Hangzhou, 310014 Zhejiang People’s Republic of China; 2 College of Basic Medical Sciences, Second Military Medical University, Shanghai, China

**Keywords:** miR-21, Non-small cell lung cancer cells, CD36, Fatty acid metabolism, Migration, Cell growth

## Abstract

**Background:**

Lung cancer is one of the most common malignant tumors worldwide. CD36 is a receptor for fatty acids and plays an important role in regulating fatty acid metabolism, which is closely related to tumorigenesis and development. The regulation of miR-21 and its role in tumorigenesis have been extensively studied in recent years. However, the relationship between miR-21 and CD36 regulated fatty acid metabolism in human non-small cell lung cancer remains unknown.

**Methods:**

In this study, lentivirus transfection, qRT-PCR, cell migration, immunofluorescence, and western blot were used to examine the relationship between miR-21 and CD36 regulated fatty acid metabolism and the regulation role of miR-21 in human non-small cell lung cancer.

**Results:**

This study demonstrated that up-regulation of miR-21 promoted cell migration and cell growth in human non-small cell lung cancer cells. Moreover, the intracellular contents of lipids including cellular content of phospholipids, neutral lipids content, cellular content of triglycerides were significantly increased following miR-21 mimic treatment compared with control, and the levels of key lipid metabolic enzymes FASN, ACC1 and FABP5 were obviously enhanced in human non-small cell lung cancer cells. Furthermore, down-regulation of CD36 suppressed miR-21 regulated cell growth, migration and intracellular contents of lipids in human non-small cell lung cancer cells, which suggested that miR-21 promoted cell growth and migration of human non-small cell lung cancer cells through CD36 mediated fatty acid metabolism. Inhibition of miR-21 was revealed to inhibit cell growth, migration, intracellular contents of lipids, and CD36 protein expression level in human non-small cell lung cancer cells. In addition, PPARGC1B was a direct target of miR-21, and down-regulation of PPARGC1B reversed the inhibition of CD36 expression induced by miR-21 inhibitor.

**Conclusions:**

These results explored the mechanism of miR-21 promoted non-small cell lung cancer and might provide a novel therapeutic method in treating non-small cell lung cancer in clinic.

## Background

Lung cancer is one of the most common malignant tumors worldwide [1]. The survival rate of patients with early lung carcinoma is higher. However, the prognosis can be significantly worse once metastasis occurs [2]. The 5-year survival rate for advanced metastatic lung cancer does not exceed 10% [3]. Despite the development of new drugs and new treatments in the field of lung cancer in recent years, the extremely low 5-year survival and side effects of lung cancer patients still exist [4]. Therefore, exploring new molecular targets and mechanisms of action in the progression of lung cancer is essential for inhibiting the growth of lung cancer cells and prolonging the 5-year survival of lung cancer patients [5]. microRNAs (miRNAs) are a class of RNA molecules that are about 21 to 23 nucleotides in length and are widely found in eukaryotes. miRNAs can bind to the 3′ untranslated region of their downstream target genes and regulate the expression of other genes [6]. miRNAs are closely related to the occurrence of various tumors, and can play an important role in tumor cell proliferation, apoptosis, differentiation, and metastasis by regulating target mRNA [7, 8]. miR-21 is one of the miRNAs that promotes cancer progression and targets multiple tumor suppressor genes involved in proliferation, apoptosis and invasion [9, 10]. The regulation of miR-21 and its role in tumorigenesis have been extensively studied in recent years [11, 12]. A number studies have found the elevated expression of miR-21 in various cancer cells [13, 14]. Previous study has shown that miR-21 enhances drug-resistant lung adenocarcinoma cancer cell invasion and migration through targeting HBP1 [15]. However, the detailed mechanisms of miR-21 regulated human non-small cell lung cancer remain to solve.

Lipids mainly include fatty acids (FA), triglycerides and cholesterol, which are important sources of energy metabolism in the body [16]. Metabolites of fatty acids are involved in the regulation of the expression of a variety of genes [17, 18]. Recent studies have shown that many fatty acid metabolic pathways have changed in various cancer cells [19, 20]. In the process of cancer development, changes in fatty acid metabolism pathways not only provide energy for the occurrence and development of cancer, but also play an important role in biofilm macromolecules and signaling molecules [21]. Previous study demonstrated that LNMICC accelerated metastasis by reprogramming fatty acid metabolism in cervical cancer [22]. CD36 is a receptor for lipids and fatty acids and plays an important role in metabolic syndrome and related cardiac activities, which involved in lipid metabolism, long-chain fatty acid adsorption, apoptotic residue clearance, and macrophage phagocytosis [23–25]. Studies have found that CD36-regulated fatty acid metabolism is closely related to tumorigenesis and development [26, 27]. Gloria Pascual et al. found the ability of fatty acid receptor CD36 and lipid metabolism genes, in initiating metastasis, and palmitic acid or a high-fat diet enhanced the metastatic potential of CD36^+^ metastasis-initiating cells in a CD36-dependent manner [28].

Khaidakov et al. demonstrated that Oxidized LDL enhanced upregulation of proliferative and pro-inflammatory signaling in human breast mammary epithelial cells, which involved in stimulation of miR-21 expression, as well as upregulation of CD36 expression [29]. These results suggested that miR-21 might positively correlated with CD36 in human breast mammary epithelial cells. A recent study has shown that lipids could promote the growth of breast cancer cells via regulation of CD36 expression [30]. On the other hand, Calo et al. Revealed that miR-21 deletion in hepatocytes increases modulated the expression of multiple key metabolic transcription factors involved in fatty acid uptake, de novo lipogenesis, gluconeogenesis and glucose output [31]. However, the relationship between miR-21 and CD36 regulated fatty acid metabolism in human non-small cell lung cancer remains unknown.

In this study, we focused the effect of miR-21 on lung cancer cell growth and migration and the tentative relation to CD36 regulated lipid metabolism. We found miR-21 enhanced cell growth, migration, intracellular contents of lipids and key lipid metabolic enzymes in human non-small cell lung cancer cells. Moreover, down-regulation of CD36 suppressed miR-21 regulated cell growth, migration and intracellular contents of lipids in human non-small cell lung cancer cells. In addition, we also found inhibition of miR-21 suppressed cell growth, migration and intracellular contents of lipids in human non-small cell lung cancer cells. These results explored the mechanism of miR-21 promoted non-small cell lung cancer and might provide a novel therapeutic method in treating non-small cell lung cancer in clinic.

## Materials and methods

### Cell culture

The A549 and H1703 human non-small cell lung cancer cells were obtained from the cell bank of Shanghai Institutes for Biological Sciences. Cells were cultured in RPMI-1640 medium supplemented with 100 U/mL penicillin, 100 μg/mL streptomycin and 10% fetal bovine serum at 37 °C in an atmosphere of 5% CO_2_.

### miRNA

The miR-21-3p mimic, inhibitor and the respective negative controls were obtained from GenePharma (Shanghai, China). The cell transfection was performed using Lipofectamine 2000 Reagent (Invitrogen) according to the manufacturer’s instruction. The cell number was determined after different treatments.

### Plasmid, lentivirus construction, and transfection

Oligonucleotides of shRNA for CD36 and PPARGC1B were obtained from Sangon Biotech (Shanghai). After co-transfection, the virus media were harvested. Cells were infected for 72 h with lentivirus contained CD36 and control shRNA respectively. A549 cells were seeded in 6-well plate overnight, and then washed these cells with PBS for three times and these cells were finally both received lentivirus containing CD36 or control shRNA. The sequence of shRNA was as follows: CD36, TTGTACCTATACTGTGGCTAAATGAGAC. PPARGC1B, GAGTGCGAGGTGC TGACAAGAAATAGGA.

### Quantitative real-time reverse transcription polymerase chain reaction (qRT-PCR)

Trizol Reagent (Invitrogen) was used to extract RNA from A549 cell lines. An ABI PRISM 7500 real-time PCR System and SYBR Green Master Mix (Roche, Shanghai, China) were used to perform polymerase chain reactions. β-actin and U6 were used as the internal references for mRNA and miRNA respectively. PCR primers were as follows, miR-21 forward: 5′-GGACTAGCTTATCAGACTG-3′, reverse: 5′-CATC AGATGCGTTGCGTA-3′; CD36 forward: 5′-GAACCACTGCTTTCAAAA ACTGG-3′, reverse: 5′-GTCCTGAGTTATATTTTCCTTGG-3′; PPARGC1B forward: 5′-GAACTTGACCTCTCCCAGCT-3′, reverse: 5′-AGGGCCTCATTCTC ACTGTC-3′; U6 forward: 5′-GC TTCGGCAGCACATATACTAAAAT-3′, reverse: 5′-CGCTTCACGAATTTGCGTGTCAT-3′; GAPDH forward: 5′-ATCTGGAGTTTACCGCTGG-3′, reverse: 5′-TACCG ATGTCTGGTAGACGA T-3′.

### Cell migration

Wound-healing assays were performed to evaluate cell migration ability. Cells were transfected with miR-21 mimic or control in culture medium containing 2% FBS. Wound closure was observed at 0 h, 24 h, and 48 h and photographed using a microscope (Olympus, Tokyo, Japan) after scratch at the bottom of the plates. The migration distance of wound closure was calculated between each group.

### Western blot

After transfection, cells were treated in a lysis buffer containing a complete protease inhibitor cocktail (Shanghai Roche Pharmaceuticals), followed by the extraction and quantification of proteins. Subsequently, the protein was transferred the into nitrocellulose membranes, blocked, and incubated in specific primary antibody at 4 °C overnight. The membranes were washed and incubated with second antibody for 2 h at room temperature. Finally, the protein bands were visualized using chemiluminescence detection system. The following antibodies were used: anti- FASN (Abcam, UK), anti- ACC1 (Cell Signaling Technology, Danvers, MA, USA), anti-FABP5 (Cell Signaling Technology, Danvers, MA, USA); anti-CD36 (Santa CruzBiotechnology, Santa Cruz, CA, USA).

### Quantification of neutral lipid

The lipophilic fluorescence dye BODIPY 493/503 (Invitrogen) was employed for monitoring the neutral lipid accumulation in A549 cells according to the manufacturer’s instructions. Briefly, cells were washed in Phosphate Buffered Saline (PBS), fixed with 4% paraformaldehyde and stained with BODIPY 493/503 (1 μg/mL) for 45 min at room temperature, and then nuclei were counterstained with Hoechst for 15 min. The results of immune staining were detected using a fluorescence microscope (Olympus, Tokyo, Japan).

### Quantification of phospholipids and triglycerides

Intracellular phospholipids and triglycerides content were assayed by EnzyChrom™ phospholipids assay kit (BioAssay Systems, Hayward, CA, USA) and EnzyChrom™ triglycerides assay kit (BioAssay Systems, Hayward, CA, USA) respectively, according to the procedure provided in the kit.

### Dual luciferase assays

The fragments contain the wild type (wt) and the mutant type of PPARGC1B gene were respectively cloned into the pGL3 luciferase miRNA target expression vector (Promega, Madison, Wisconsin). And then the recombinant reporter vector named pGL3-PPARGC1B-wt or pGL3-PPARGC1B-mut were synthesized. The reporter vector pGL3-PPARGC1B-wt/pGL3-PPARGC1B-mut and miR-21 mimic were co-transfected into A549 cells with Lipofectamine 2000 (Invitrogen). The luciferase activity of the cells were detected using a Dual Luciferase Reporter Assay System (Promega).

### Statistical analysis

The data were analyzed using SPSS 17.0 software (SPSS Inc., Chicago, IL, USA). The data were presented as the mean ± SD of at least three independent experiments. The independent sample *t* test was used for comparing groups for statistical differences. Statistical significance was defined as P < 0.05.

## Results

### miR-21 enhanced cell growth and migration in human non-small cell lung cancer cells

**Fig. 1:**
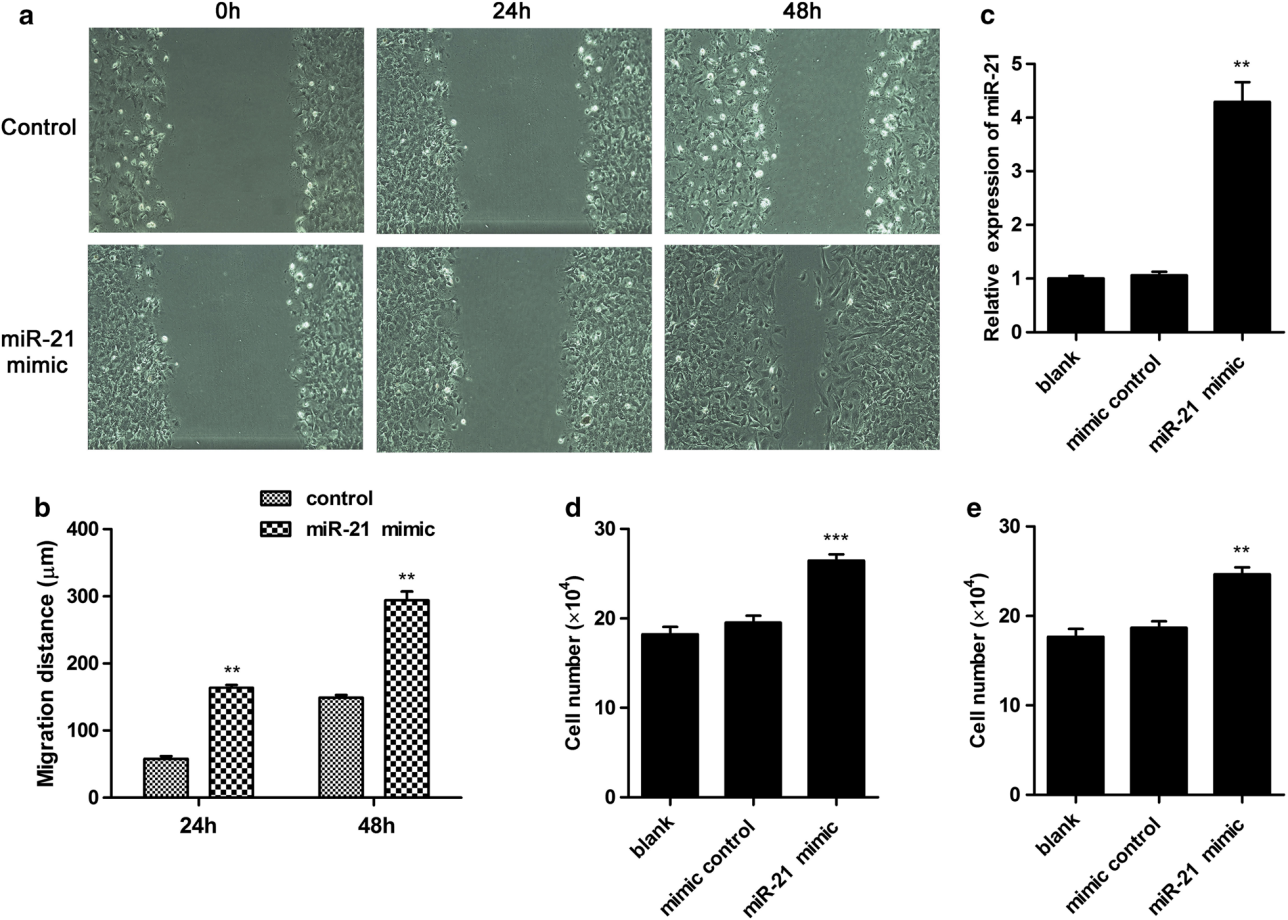
miR-21 enhanced growth and migration in human non-small cell lung cancer cells. **a** and **b** miR-21 enhanced cell migration in A549 cells. Cells were transfected with miR-21 mimic or mimic control. At 24 h or 48 h after transfection, migration abilities of cells were detected. **c** miR-21 mimic transfection enhanced miR-21 expression level. A549 cells were transfected with miR-21 mimic or mimic control. The miR-21 expression level was detected. A549 cells (**d**) or H1703 cells (**e**) were transfected with miR-21 mimic or mimic control. Cell number was counted after trypan blue staining. **P < 0.01, ***P < 0.001

To investigate the effect of miR-21 on human non-small cell lung cancer cells, miR-21 mimic was constructed and treated to A549 cells. The transfection efficiency of miR-21 mimic in A549 cells were determined. The result revealed miR-21 mimic treatment up-regulated more than fourfolds of miR-21 expression (Fig. 1c). To further explore the effect of miR-21 on cell migration ability, A549 cells were transfected with or without miR-21 mimic for 24 h and 48 h. The results exhibited miR-21 mimic treatment significantly enhanced cell migration abilities compared with control (Fig. 1a, b). Moreover, the cell growth was significantly increased following miR-21 mimic treatment compared with control in A549 cells and H1703 cells (Fig. 1d, e). These results suggested that miR-21 enhanced cell growth and migration in human non-small cell lung cancer cells.

### miR-21 enhanced the intracellular contents of lipids and key lipid metabolic enzymes in human non-small cell lung cancer cells

**Fig. 2:**
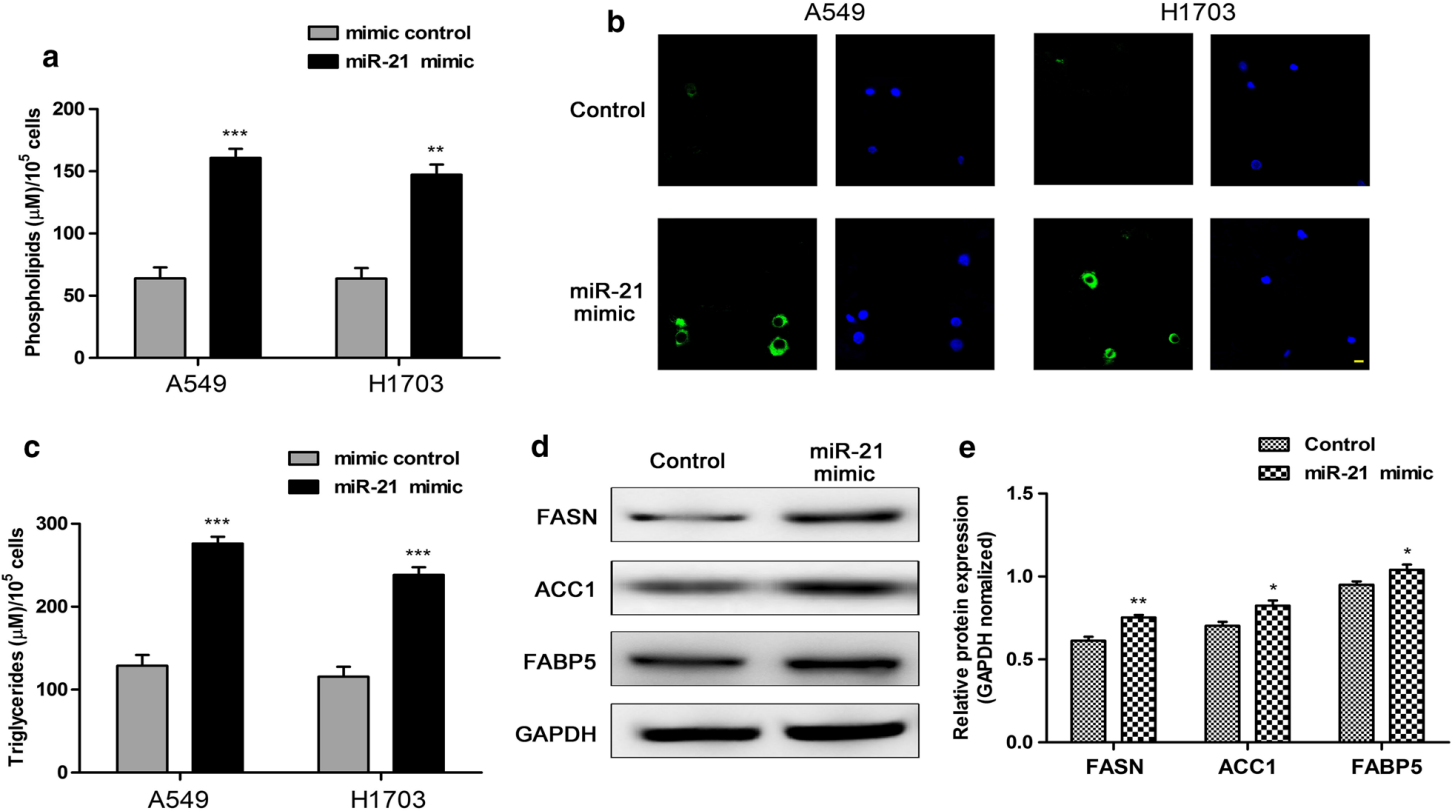
miR-21 enhanced the intracellular contents of lipids and key lipid metabolic enzymes in human non-small cell lung cancer cells. **a** Cellular content of phospholipids was detected in human non-small cell lung cancer cells. **b** The neutral lipids content was detected by staining with BODIPY 493/503 dye and DAPI in human non-small cell lung cancer cells. The neutral lipids content was stained with BODIPY 493/503 (green) and nuclei were stained with DAPI (blue). Scale bar: 10 μm. **c** Cellular content of triglycerides was detected in human non-small cell lung cancer cells. **d**, **e** The protein expression levels and quantifications of key lipid metabolic enzymes FASN, ACC1 and FABP5 were detected in A549 cells. **P < 0.01, ***P < 0.001

To investigate the tentative mechanism of miR-21 regulated human non-small cell lung cancer cells, the intracellular contents of lipids and key lipid metabolic enzymes were determined following miR-21 mimic treatment. The result demonstrated that miR-21 mimic treatment increased nearly threefolds of cellular phospholipids compared with control in A549 cells or H1703 cells (Fig. 2a). Moreover, the neutral lipids content was detected by staining with BODIPY 493/503 dye and DAPI in human non-small cell lung cancer cells. The result showed that miR-21 mimic treatment obviously promoted the neutral lipids content in A549 and H1703 cells (Fig. 2b). Furthermore, cellular content of triglycerides was significantly increased following miR-21 mimic treatment compared with control in human non-small cell lung cancer cells (Fig. 2c). In addition, to further explore the effect of miR-21 on fatty acid metabolism, the protein expression levels of key lipid metabolic enzymes FASN, ACC1 and FABP5 were detected and the result revealed that miR-21 mimic treatment apparently promoted the expression levels of key lipid metabolic enzymes in A549 cells (Fig. 2d, e). These results suggested that miR-21 enhanced the intracellular contents of lipids and key lipid metabolic enzymes in human non-small cell lung cancer cells.

### Down-regulation of CD36 suppressed miR-21 regulated cell growth, migration and intracellular contents of lipids in human non-small cell lung cancer cells

**Fig. 3:**
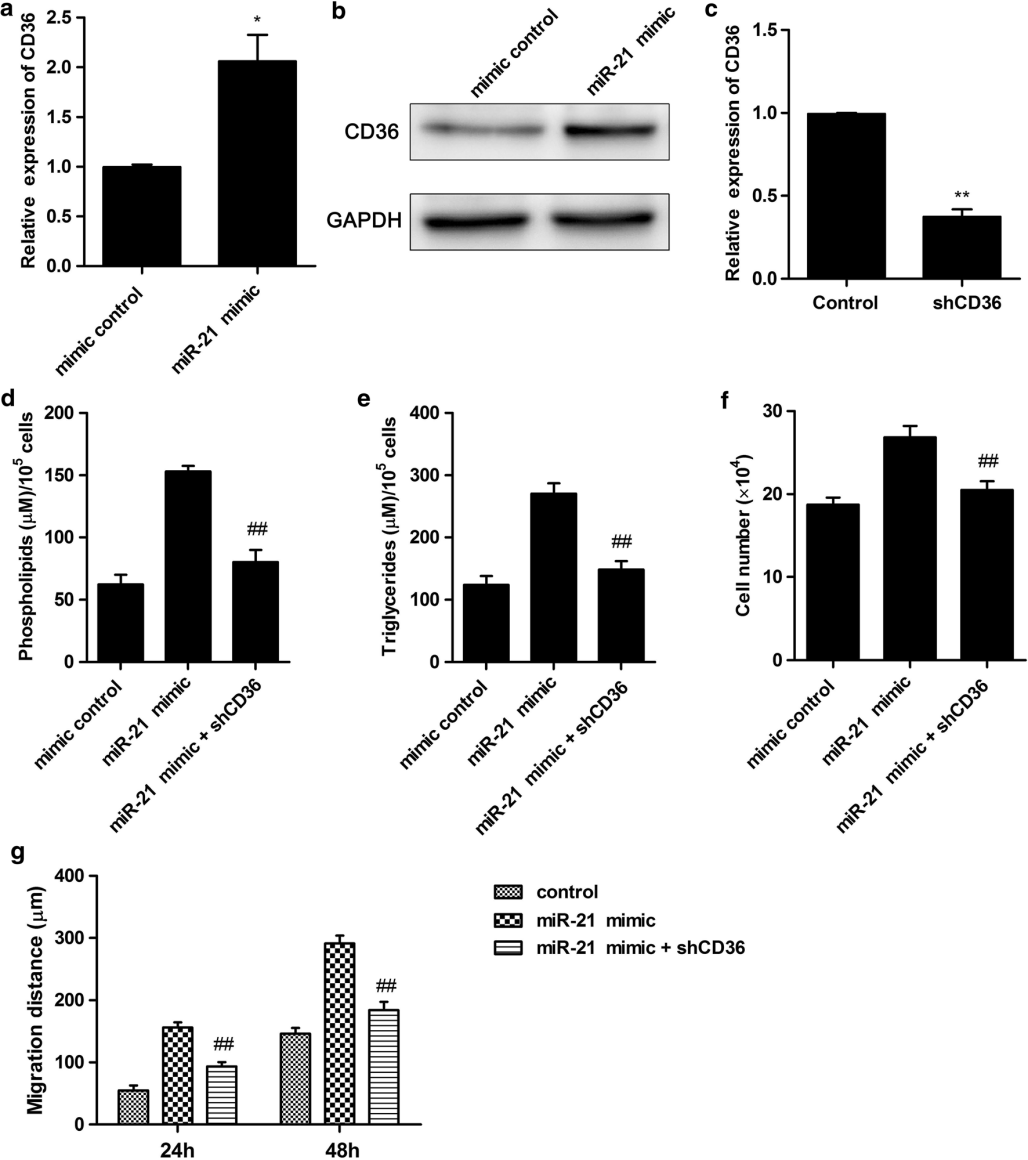
Down-regulation of CD36 inhibited miR-21 induced cell growth, migration and intracellular contents of lipids in human non-small cell lung cancer cells. **a** miR-21 mimic increased CD36 expression level. Cells were treated with miR-21 mimic or mimic control. The CD36 expression level was detected. **b** The protein expression level of CD36 was detected. **c** Cells were treated with shCD36 or control, The CD36 expression level was detected. **d** Cellular content of phospholipids was detected in human non-small cell lung cancer cells. **e** Cellular content of triglycerides was detected in human non-small cell lung cancer cells. **f** Cells were transfected with miR-21 mimic and/or shCD36. Cell number was counted after trypan blue staining. **g** Cells were treated with miR-21 and/or shCD36 for 24 h or 48 h. Migration abilities of cells were detected. *P < 0.05 VS control, **P < 0.01 VS control, ^##^P < 0.01 VS miR-21 mimic

To further investigate the effect of miR-21 on fatty acid metabolism, the key receptor CD36 in the upstream of fatty acid metabolism was detected. The results demonstrated that miR-21 mimic increased CD36 expression level compared with control, and obviously promoted CD36 protein expression level (Fig. 3a, b). To investigate the effect of CD36 on miR-21 regulated cell growth, migration and intracellular contents of lipids in human non-small cell lung cancer cells, CD36 was subsequently knockdown by transfection with lentivirus shRNA. After transfection with CD36 shRNA (shCD36), the expression level of CD36 was significantly decreased compared with control (Fig. 3c). Moreover, cellular content of phospholipids and triglycerides were significantly decreased after transfection with shCD36 compared with miR-21 mimic treatment (Fig. 3d, e). In addition, the cell number and migration ability were also significantly down-regulated compared with miR-21 mimic treatment (Fig. 3f, g). These results suggested that miR-21 promoted cell growth, migration and fatty acid metabolism through regulating CD36 expression.

### Inhibition of miR-21 suppressed cell growth, migration and intracellular contents of lipids in human non-small cell lung cancer cells

**Fig. 4:**
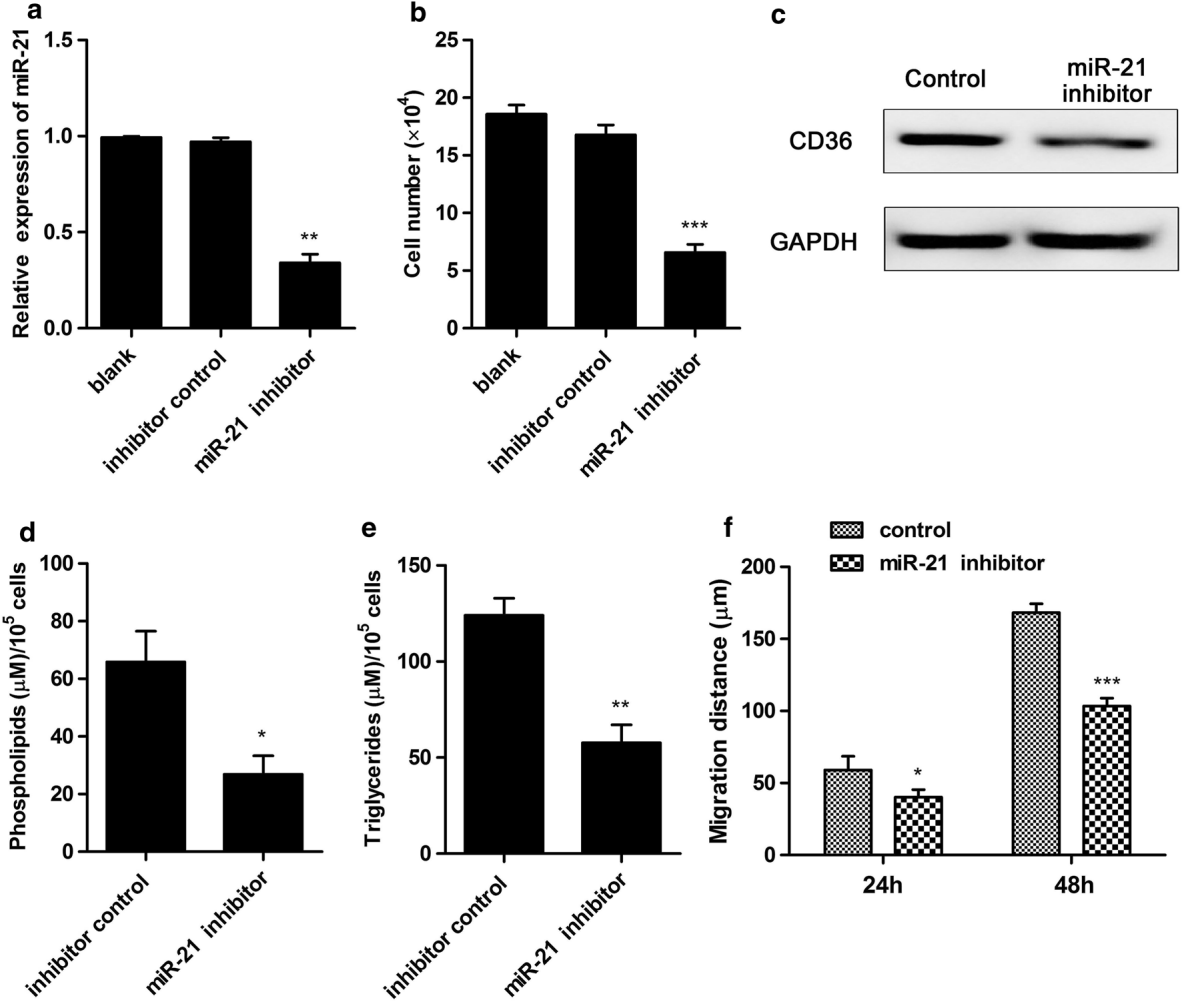
Inhibition of miR-21 suppressed cell growth, migration and intracellular contents of lipids in human non-small cell lung cancer cells. **a** miR-21 inhibitor treatment inhibited miR-21 expression level. Cells were treated with miR-21 inhibitor or inhibitor control. The miR-21 expression level was detected. **b** miR-21 inhibitor suppressed cell growth. Cells were transfected with miR-21 inhibitor or inhibitor control. Cell number was counted after trypan blue staining. **c** The protein expression level of CD36 was detected. **d** Cellular content of phospholipids was detected in human non-small cell lung cancer cells. **e** Cellular content of triglycerides was detected in human non-small cell lung cancer cells. **f** miR-21 inhibitor suppressed cell migration. Cells were treated with miR-21 inhibitor or inhibitor control for 24 h or 48 h. migration abilities of cells were detected. *P < 0.05, **P < 0.01, ***P < 0.001

To further examined the effect of miR-21 on cell growth, migration, and fatty acid metabolism, miR-21 inhibitor was constructed and treated to A549 cells. As expected, miR-21 inhibitor treatment significantly decreased miR-21 expression level compared with control (Fig. 4a). Moreover, the cell number and migration ability were significantly down-regulated compared with control (Fig. 4b, f). Furthermore, cellular content of phospholipids was slightly decreased, and cellular content of triglycerides was significantly decreased after transfection with miR-21 inhibitor compared with control (Fig. 4d, e). In addition, miR-21 inhibitor treatment also obviously decreased CD36 protein expression level compared with control in A549 cells (Fig. 4c). These results suggested that inhibition of miR-21 suppressed cell growth, migration and intracellular contents of lipids in human non-small cell lung cancer cells.

### PPARGC1B was involved in miR-21 regulated CD36 expression

**Fig. 5:**
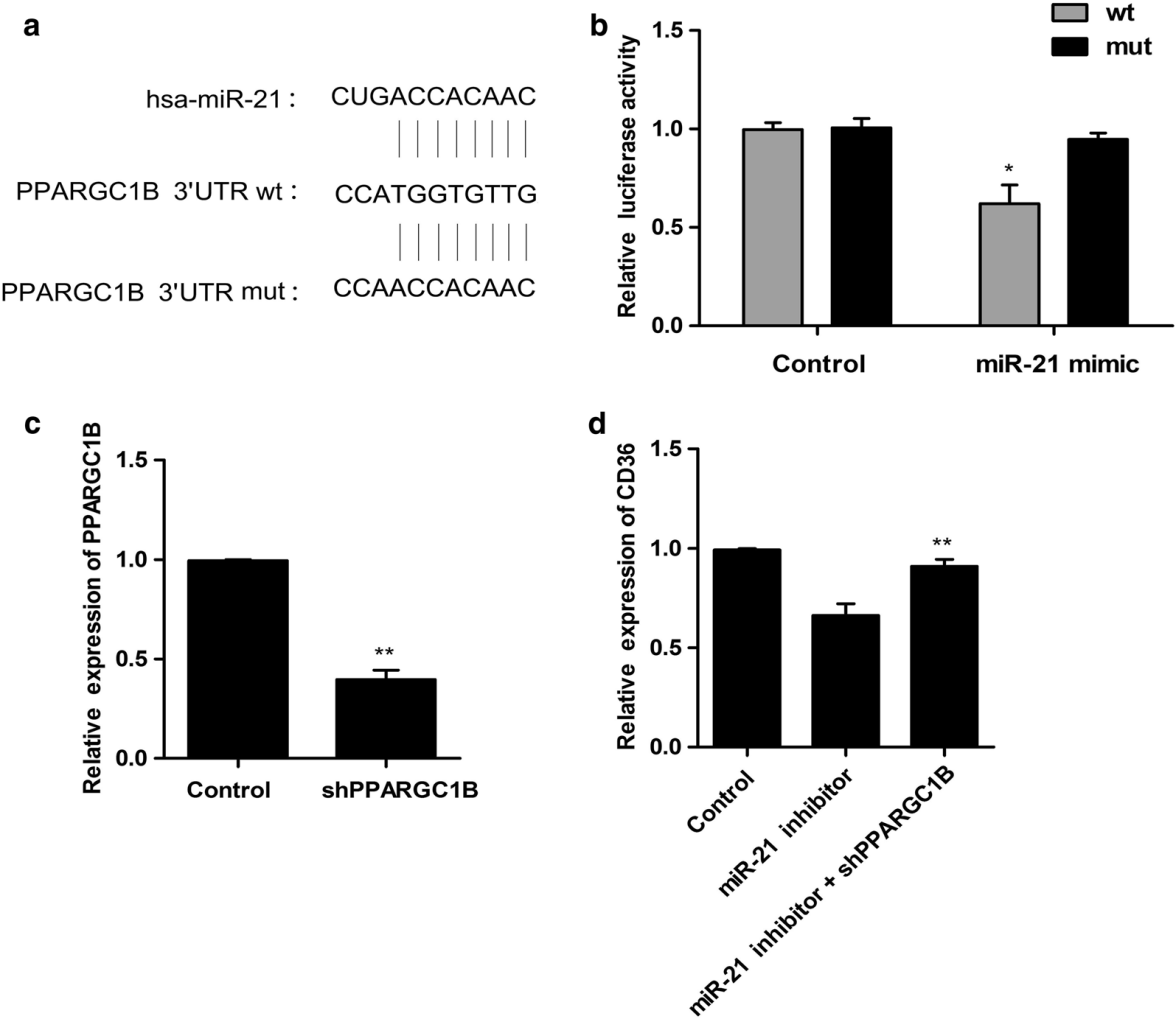
PPARGC1B was involved in miR-21 regulated CD36 expression. **a** The predicted miR-21 targeting site is present at nucleotide of the 3′ UTR of PPARGC1B. wt, wild-type reporter construct containing PPARGC1B 3′UTR; mut, establishes with mutations in relevant regions; **b** miR-21 mimic treatment decreased the luciferase activity of PPARGC1B wt, but not mut. **c** Cells were treated with shPPARGC1B or control, The PPARGC1B expression level was detected. **d** shPPARGC1B treatment reversed miR-21 inhibitor decreased CD36 expression level. Cells were treated with miR-21 inhibitor or combined shPPARGC1B. The CD36 expression level was detected. *P < 0.05, **P < 0.01

To investigate the detailed mechanism of miR-21 regulated fatty acid metabolism, miR-21 was predicted to have the binding site with the 3′UTR of PPARGC1B (Fig. 5a). PPARGC1B was known to play an important role in multiple metabolic process. To investigate the relationship between miR-21 and PPARGC1B, dual luciferase assay was conducted in A549 cells. The luciferase activities were significantly decreased by treatment with pGL3-PPARGC1B-wt and miR-21 mimic. However, the luciferase activity in cells transfected with pGL3-PPARGC1B-mut revealed no significant difference following treatment of miR-21 mimic (Fig. 5b). Next, to further examine the effect of PPARGC1B on miR-21 regulated CD36 expression, PPARGC1B was down-regulation by shRNA and the efficiency was determined in Fig. 5c. The relative expression of CD36 was decreased by the treatment of miR-21 inhibitor. However, after combined treatment with miR-21 inhibitor and shPPARGC1B, the relative expression of CD36 was significantly reversed compared with miR-21 inhibitor treatment group (Fig. 5d). These results suggested that PPARGC1B might involve in miR-21 regulated CD36 expression.

## Discussion

Abnormal lipid metabolism and related pathologies are associated with cd36-mediated signal transduction dysfunction [32]. Several studies have found that fatty acid receptor CD36 is highly expressed in many types of tumors [28, 33]. Previous study has shown that a kind of cells expressing high level of the fatty acid receptor CD36 has been found in human oral cancer samples, and they have a high metastatic potential in mice [34]. miRNAs play an important regulatory role in post-transcriptional regulation of gene expression, and participate in a variety of biological processes including intercellular signal transmission, cell proliferation, growth, differentiation, apoptosis, and cell metabolism [35]. Recent studies have found that miRNA plays an important role in energy metabolism such as glucose metabolism, fat metabolism and amino acid metabolism [36, 37]. Specific miRNA regulates lipid metabolism by binding to the 3′UTR of the related lipid metabolism regulating gene. Cruz-Gil et al. found miR-19b-1 suppressed invasion in colon cancer cells by targeting the lipid metabolic axis ACSL/SCD [38]. Cheng et al. demonstrated that down-regulation of miR-148a was revealed to be correlated with poor clinical outcomes in hepatocellular carcinoma (HCC) patients, and miR-148a deletion accelerated hepatocarcinogenesis through promoting lipid metabolic disorders in mice [39].

Recently, the role of miRNAs in the regulation of various tumor lipids is gradually elucidated [40]. However, whether miRNAs regulate fatty acid metabolism in non-small cell lung cancer, and its role in the progression of non-small cell lung cancer remain unclear. Here, in this study, we found miR-21 mimic treatment significantly enhanced cell migration abilities and cell growth compared with control in human non-small cell lung cancer cells. Moreover, to investigate the effect of miR-21 on fatty acid metabolism in human non-small cell lung cancer cells, the intracellular contents of lipids and key lipid metabolic enzymes were determined and the results revealed cellular content of phospholipids, neutral lipids content, cellular content of triglycerides, and the levels of key lipid metabolic enzymes FASN, ACC1 and FABP5 were significantly increased following miR-21 mimic treatment compared with control in A549 cells. Furthermore, CD36 was subsequently knockdown by transfection with lentivirus shRNA to explore the mechanism of miR-21 regulated fatty acid metabolism. The results demonstrated that down-regulation of CD36 suppressed miR-21 regulated cell growth, migration and intracellular contents of lipids in human non-small cell lung cancer cells, which suggested that miR-21 promoted cell growth, migration and fatty acid metabolism through up-regulated CD36 expression. In addition, we also found inhibition of miR-21 suppressed cell growth, migration, intracellular contents of lipids, and CD36 protein expression level in human non-small cell lung cancer cells. In order to investigate the detailed mechanism of miR-21 regulated CD36 expression, PPARGC1B was revealed to be a direct target of miR-21, and down-regulation of PPARGC1B reversed the inhibition of CD36 expression induced by miR-21 inhibitor. Although PPARGC1B was revealed to involve in miR-21 regulated CD36 expression, the mechanism of PPARGC1B in miR-21 regulated fatty acid metabolism needed further explore.

## Conclusion

This study demonstrated that up-regulation of miR-21 promoted cell migration and cell growth in human non-small cell lung cancer cells. Moreover, the intracellular contents of lipids including cellular content of phospholipids, neutral lipids content, cellular content of triglycerides were significantly increased following miR-21 mimic treatment compared with control, and the levels of key lipid metabolic enzymes FASN, ACC1 and FABP5 were obviously enhanced in human non-small cell lung cancer cells. Furthermore, down-regulation of CD36 suppressed miR-21 regulated cell growth, migration and intracellular contents of lipids in human non-small cell lung cancer cells, which suggested that miR-21 promoted cell growth and migration of human non-small cell lung cancer cells through CD36 mediated fatty acid metabolism. In addition, inhibition of miR-21 was revealed to inhibit cell growth, migration, intracellular contents of lipids, and CD36 protein expression level in human non-small cell lung cancer cells. The results also demonstrated that PPARGC1B was a direct target of miR-21, and down-regulation of PPARGC1B reversed the inhibition of CD36 expression induced by miR-21 inhibitor. These results explored the mechanism of miR-21 promoted non-small cell lung cancer and might provide a novel therapeutic method in treating non-small cell lung cancer in clinic.

## Data Availability

All data generated or analyzed during this study are included in this published article.
